# Exogenous Antioxidants Impact on UV-Induced Changes in Membrane Phospholipids and the Effectiveness of the Endocannabinoid System in Human Skin Cells

**DOI:** 10.3390/antiox10081260

**Published:** 2021-08-06

**Authors:** Agnieszka Gęgotek, Anna Jastrząb, Marta Dobrzyńska, Michał Biernacki, Elżbieta Skrzydlewska

**Affiliations:** Department of Analytical Chemistry, Medical University of Bialystok, 15-089 Bialystok, Poland; anna.jastrzab@umb.edu.pl (A.J.); domarta16@gmail.com (M.D.); michal.biernacki@umb.edu.pl (M.B.); elzbieta.skrzydlewska@umb.edu.pl (E.S.)

**Keywords:** keratinocytes, skin fibroblasts, UV radiation, rutin, ascorbic acid, endocannabinoids, phospholipid peroxidation, phospholipid fatty acids, Zeta potential

## Abstract

Natural antioxidants effectively counteract changes caused by UV radiation in human skin cells. However, their action is limited due to their lipo/hydrophilicity. Therefore, the aim of this study was to analyze the mutual protective action of hydrophilic ascorbic acid and partially lipophilic rutin against UVA/UVB-induced changes in membranes phospholipid and endocannabinoid system in keratinocytes and fibroblasts. Obtained results clearly showed that, despite the stronger antioxidant properties of ascorbic acid, the lipid membranes were more effectively protected against UV-induced oxidation by rutin, including changes in phospholipid fatty acid levels, prevention against reactive aldehydes formation and endocannabinoids degradation. Ascorbic acid more strongly prevented UV-induced endocannabinoid receptors expression in fibroblasts, especially CB1. However, the combined action of used antioxidants resulted in the greatest cytoprotective effect, which was evident in the inflammatory marker TNFα down-regulation and increased cell viability following cell irradiation. The applied mixture of antioxidants showed a stronger protective in relation to membrane phospholipids in keratinocytes and in the endocannabinoid system in fibroblasts. In conclusion, it can be suggested that combined antioxidant capacities of ascorbic acid and rutin protects against lipid peroxidation but also decreases the UV-induced inflammation by direct interaction with the endocannabinoid system, thus increasing skin cell viability.

## 1. Introduction

Human skin, as the most external structure of the body, constitutes the basic protection of internal organs by creating a barrier against the harmful factors of the external environment and, at the same time, provides a direct link with this environment. Cells creating skin are arranged in three main layers: epidermis made up mainly of keratinocytes, dermis built predominantly by the fibroblasts, and subcutaneous tissue (hypodermis) [1]. Due to the role of skin cells in the maintenance of the whole organism homeostasis, they are characterized by the well-developed protective, including antioxidant, and self-repairing system. Skin fibroblasts and keratinocytes are rich in enzymatic protective systems; however, their activation is often dependent on signaling molecules belonging to the lipid metabolites [2]. That is especially visible in the UV-irradiated skin cells, where radiation activates the antioxidant system and induces inflammatory reaction through an enhanced level of lipid peroxidation products, including reactive aldehydes and isoprostanes [3]. It deserves special attention, especially since UV radiation contained in sunlight is one of the most common harmful factors to which human skin is exposed [4]. Products of the oxidative lipids metabolism, especially small molecular aldehydes, have been described as one of the most reactive and, therefore, are the fastest signals transducers that can modify various biologically important pathways [5]. Moreover, UV radiation has a strong effect on the endocannabinoid system in the skin fibroblasts and keratinocytes, which includes both decreases in basal endocannabinoid (anandamide, 2-AG) levels as well as enhanced expression of their main membrane receptors (CB1/2, TVRP1) [3,6]. So far, the direct role of endocannabinoids in human skin has been described in the case of wound healing [7], but also under neo-plastic processes [8], where its increased level promoted cell proliferation and has an antiapoptotic effect. However, their basic action concerns the interactions with specific receptors [9]. It is known that CB1 activation leads to oxidative cell reactions, while activated CB2 induces antioxidant and anti-inflammatory properties [10]. On the other hand, activation of CB1–CB2 heterodimers block the effect of CB1/2 antagonists, as well as agonists, and results in the phosphorylation of mitogen activated kinases (MAPK), including ERK1/2, as well as Akt kinases, which leads to the phosphorylation and activation of transcription factor Nrf2 [11,12]. Next, Nrf2 is translocated into the nucleus, where it initiates the expression of cytoprotective proteins, including antioxidant enzymes [13]. This process is considered to be a positive self-defense reaction of skin cells to UV radiation; however, uncontrolled and continuous activation of Nrf2 factor is one of the basic features of neoplastic transformation [14]. Therefore, there is constant looking for the compounds protecting skin cells against UV-induced changes, not only with antioxidant and anti-inflammatory properties, but also with potential effects on the lipid mediators and endocannabinoid system.

**Figure 1:**
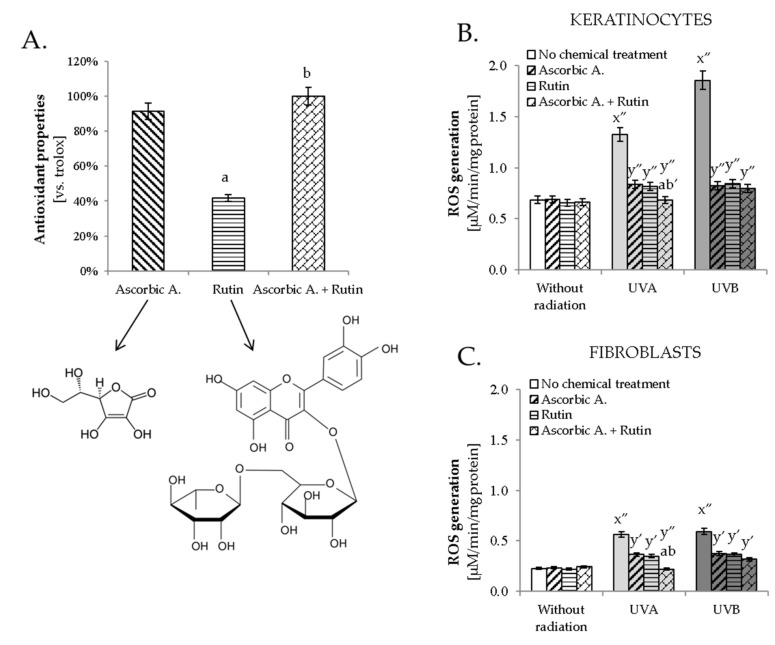
The structure and antioxidant activity of rutin (25 µM) or/and ascorbic acid (100 µM) (**A**) and their effect on reactive oxygen species (ROS) generation in keratinocytes (**B**) and fibroblasts (**C**) following exposure to UVA (30 J/cm^2^ and 20 J/cm^2^, respectively) and UVB (60 mJ/cm^2^ and 200 mJ/cm^2^, respectively) irradiation. Mean values ± SD of three independent experiments are presented. x significant differences vs. non-treated group ( x″ for *p* < 0.0005); y significant differences vs. group without chemical treatment, respectively ( y′ for *p* < 0.005, y″ for *p* < 0.0005); a significant differences vs. ascorbic acid-treated group, respectively (a for *p* < 0.05); b significant differences vs. rutin-treated group, respectively (b for *p* < 0.05; b′ for *p* < 0.005).

Examples of compounds with described effects can be found in the group of natural plant antioxidants such as rutin or ascorbic acid. Both of them are known due to their ability to scavenge free radicals, which is dependent on their chemical structure rich in electron donor groups (Figure 1A) [15]. Moreover, both of them have been described as cytoprotective compounds against UV-induced changes, and their action has been connected with protection against lipid peroxidation and endocannabinoid system activation [16,17]. However, due to the hydrophilic character of ascorbic acid and low solubility in aqueous solutions of rutin, these compounds can only protect cell ingredients in strictly narrowed areas of the cell. Therefore, more and more often, suggested methods for skincare are based on combining the action of these two compounds, which additionally promotes the observation of synergism in their operation [15,18,19,20]. Thus far, some reports have been published about the benefits of combined rutin and ascorbic acid action in relation to skin cells, which concern their mutually assisted transmembrane transport, antioxidant cooperation, proteome protection or ant-apoptotic activities [15,19,20,21]. However, despite the importance of the endocannabinoid system, the mechanism of rutin-ascorbic acid action on its activity is still not known.

Therefore, the aim of this study was to analyze and compare the combined effects of rutin and ascorbic acid on the lipid mediators and endocannabinoid system in various types of skin cells, keratinocytes and fibroblasts following UV radiation, as well as to describe the results of these effects on the cells viability and inflammatory processes.

## 2. Materials and Methods

### 2.1. Antioxidant Properties Measurement

Antioxidant properties of rutin (25 µM) or/and ascorbic acid (100 µM) were measured based on DPPH method of Tuberoso et al. [22] with minor modification. A total of 4 µL of ascorbic acid (100 µM) and/or rutin (25 µM) solution were added to 600 µL of dissolved DPPH (0.04 mM). The absorbance was measured at 517 nm after 4 min. Results were expressed versus values obtained for Trolox.

### 2.2. Cells Cultures

Human skin cells: keratinocytes (CDD 1102 KERTr) and fibroblasts (CCD 1112Sk) were obtained from American Type Culture Collection (ATCC, Manassas, VA, USA). Keratinocytes were cultured in keratinocyte-SFM medium with 1% Bovine Pituitary Extract (BPE) and human recombinant Epidermal Growth Factor (hEGF), while fibroblasts were cultured in Dulbecco′s Modified Eagle Medium (DMEM) with 10% fetal bovine serum (FBS). Both growth media were supplemented with antibiotics: penicillin (50 U/mL) and streptomycin (50 μg/mL). Cells were seeded into sterile plastic plates 100 mm diameter in a concentration 250,000 cells/plate and cultured at 37 °C in air atmosphere with 5% CO_2_. Growing medium was renewed twice a week. When cells reached 90% confluence, they were subculturing in a ratio 1:4 and 1:3, respectively, for keratinocytes and fibroblasts.

### 2.3. Cells Treatment

Keratinocytes (passage 44) and fibroblasts (passage 12) were exposed to UV radiation in cold PBS buffer (4 °C) in a distance of 15 cm from the 6 lamps (6 W each, 4.2 mW/cm^2^ and 4.08 mW/cm^2^, respectively, for 365 nm (UVA) and 312 nm (UVB); Bio-Link Crosslinker BLX 312/365; Vilber Lourmat, Eberhardzell, Germany). Totaled radiation doses were as follows: 30 J/cm^2^ and 60 mJ/cm^2^ for keratinocytes, 20 J/cm^2^ and 200 mJ/cm^2^ for fibroblasts, in the case of UVA and UVB, respectively. Exposure doses were chosen corresponding to 70% cell viability measured by the MTT test [23].

To examine the effect of ascorbic acid (Sigma-Aldrich, St. Louis, MO, USA, No. A5960) and rutin (Sigma-Aldrich, St. Louis, MO, USA, No. R2303), following exposure to UV radiation, cells were incubated 24 h in medium supplemented with ascorbic acid (100 µM) or/and rutin (25 µM). According to rutin solubility, all media contained 0.1% DMSO to maintain the same conditions for all experimental cells. Parallel control cells were incubated without irradiation in medium containing the above supplements. As a result of the experiment, 12 groups of tested keratinocytes and 12 groups of tested fibroblasts were created as follows:

a. Cells without irradiation:
  1. Control keratinocytes/fibroblasts;
  2. Keratinocytes/fibroblasts treated with ascorbic acid (100 µM);
  3. Keratinocytes/fibroblasts treated with rutin (25 µM);
  4. Keratinocytes/fibroblasts treated with ascorbic acid (100 µM) and rutin (25 µM).
b. UVA-irradiated cells:
  5. UVA-irradiated keratinocytes (30 J/cm^2^) /fibroblasts (20 J/cm^2^);
  6. UVA-irradiated keratinocytes/fibroblasts treated with ascorbic acid;
  7. UVA-irradiated keratinocytes/fibroblasts treated with rutin;
  8. UVA-irradiated keratinocytes/fibroblasts treated with ascorbic acid and rutin.
c. UVB-irradiated cells:
  9. UVB-irradiated keratinocytes (60 mJ/cm^2^) /fibroblasts (200 mJ/cm^2^);
  10. UVB-irradiated keratinocytes/fibroblasts treated with ascorbic acid;
  11. UVB-irradiated keratinocytes/fibroblasts treated with rutin;
  12. UVB-irradiated keratinocytes/fibroblasts treated with ascorbic acid and rutin.

Following incubation, cells were rinsed with cold PBS buffer (4 °C) and then scraped from the dishes. ROS generation was determined directly in living cells, and then the cells were sonicated on ice. To obtain cell lysates, sonicated cells were centrifuged for 15 min at 12,000× *g* at 4 °C to separate the membrane fraction. Supernatants were collected for analyses, and obtained data were normalized to total protein content measured using a Bradford assay [24].

### 2.4. Examination of ROS Generation

The electron spin resonance spectrometer (Noxygen GmbH/Bruker Biospin GmbH, Rheinstetten, Germany) was used for detection of intracellular ROS generation. Measurement used selective interaction of 1-hydroxy-3-methoxy-carbonyl-2,2,5,5-tetrame-thylpyrrolidine (200 µM) with ROS, resulting in stable nitroxide radicals formation, whereby accumulation according to the electron spin resonance amplitude was detected [25]. The generation of ROS was reported in micromoles per minute and normalized per milligram of protein.

### 2.5. Examination of Lipid Metabolism

#### 2.5.1. Determination of Phospholipid Fatty Acid Composition

Phospholipid fatty acids profile of keratinocytes membranes was determined according to the method of Christie [26]. Lipid components were extracted by chloroform/methanol mixture (2:1, *v*/*v)* (Folch extraction) in the presence of 0.01% butylated hydroxytoluene–BHT. Next, TLC phospholipids fatty acids were separated using a mobile phase mixture of heptane-diisoprophyl ether-acetic acid (60:40:3, *v*/*v*/*v*). After that, phospholipid fatty acids were transmethylated to fatty acid methyl esters (FAMEs) with boron trifluoride in methanol. Separation of FAMEs was performed on a capillary column coated with Varian CP-Sil88 stationary phase (50 m × 0.25 mm, ID 0.2 μm, Varian, Palo Alto, CA, USA) using GC/FID (Clarus 500 Gas Chromatograph, Perkin Elmer, MA, USA). The injection volume of the sample was 2 μL. Temperature of injector and detector was 260 °C while column temperature was programmed from 150 °C (2 min) to 230 °C (10 min) at 4 °C/min. Identification of FAMEs was performed by comparison retention time using an internal standard named 1,2-dinonadecanoyl-sn-glycero-3-phosphocholine (19:0 PC). The phospholipid fatty acid concentration was expressed as micrograms normalized per milligram of protein.

#### 2.5.2. Determination of Phospholipids Metabolizing Enzyme

Cytosolic phospholipase A2 (cPLA2) activity was measured using cPLA2 Assay Kit (Cayman Chemical Company, Ann Arbor, MI, USA, No. 765021) according to manufacturer instructions. To detect cPLA2 activity, arachidonoyl thio-PC as a substrate was used, and its hydrolysis products were detected by DTNB [27]. Enzyme-specific activity was calculated in nanomoles of free thiol released per minute normalized per milligram of protein.

#### 2.5.3. Measurement of Lipid Peroxidation Products

The level of malondialdehyde (MDA) and 4-hydroxyhexenal (4-HHE) measured by GC/MSMS [28] was estimated as a marker of lipid peroxidation. Aldehydes were converted to O-PFB-oxime-TMS derivatives derivatized by the addition of O-(2,3,4,5,6-pentafluoro-benzyl) hydroxyamine hydrochloride (0.05 M). As an internal standard, Benzaldehyde-D6 was used. Next, aldehyde derivatives were extracted with hexane and evaporated under a stream of argon gas and resuspended in N,O-bis(trimethylsilyl) trifluoroacetamide in 1% trimethylchlorosilane. Samples were injected on the column (HP-5 ms, 0.25 mm internal diameter, 0.25 µm film thickness, 30 m length) and analyzed using a 7890A GC–7000 quadrupole MS/MS (Agilent Technologies, Palo Alto, CA, USA). Derivatized aldehydes were detected in the selected ion monitoring mode. The ions used were: *m*/*z* 204.0 and 178.0 for MDA-PFB, *m*/*z* 352.0 and 226.0 for 4-HHE-PFB-TMS and *m*/*z* 307.0 for IS derivatives. Obtained concentrations were expressed as nanomoles normalized per milligram of protein.

### 2.6. Examination of Endocannabinoid System

#### 2.6.1. Endocannabinoids Level Analysis

Anandamide (AEA) and 2-arachidonoylglycerol (2-AG) level was measured using ultrahigh performance liquid chromatography-tandem mass spectrometry (UPLC-MS/MS) [29]. Octadeuterated endocannabinoids: AEA-d8 and 2-AG-d8 were used as internal standards, and all cannabinoids from samples were isolated using solid-phase extraction (SPE). Analysis was performed using an Agilent 1290 UPLC system with a Zorbax Extend C18 column (2.1 mm × 150 mm, 1.8 mm, Agilent, Santa Clara, CA, USA) and interfaced with an Agilent 6460 triple quadrupole mass spectrometer with electrospray ionization source (ESI). The samples were analyzed in positive-ion mode using multiple reaction monitoring (MRM). Transition of the precursor to the product ion was: *m*/*z* 348.3→62.1 for AEA, and *m*/*z* 379.3→287.2 for 2-AG. Endocannabinoids concentrations were expressed as femtomoles normalized per milligram of protein.

#### 2.6.2. Determination of Endocannabinoids Metabolizing Enzymes

FAAH (fatty acid amide hydrolase) (EC.3.5.1.99) activity was determined according to the Siegmund procedure [30] based on the spectrophotometric measurement of m-nitroaniline (m-NA) releasing from decanoyl m-nitroaniline. Enzyme activity was expressed as the amount of enzyme metabolizing 1 pmol of substrate per minute normalized per milligram of protein.

MAGL (monoacylglycerol lipase) (EC.3.1.1.23) activity was analyzed basing on spectrophotometric determination of 5′-thio-2-nitrobenzoic acid releasing from arachidonoyl-1-thio-glycerol [31]. Enzyme activity was expressed as the amount of enzyme metabolizing 1 pmol of substrate per minute normalized per milligram of protein.

#### 2.6.3. Determination of Cannabinoids Receptors

Endocannabinoid receptors CB1 and CB2 expression were analyzed using Western blot technique described below.

### 2.7. Examination of Protein Expression

Protein expression was determined by performing Western blot analysis [32]. Whole-cell lysates or membrane fractions were separated by 10% Tris-Glycine SDS-PAGE. Following proteins electrophoretic transfer into nitrocellulose membrane, blots were blocked with 5% skim milk in TBS-T buffer (5% Tween 20) for 1 h. Primary monoclonal antibodies against TNFα, β-actin, Na^+^K^+^ATPase (Sigma-Aldrich, St. Louis, MO, USA), and CB1 and CB2 (Santa Cruse Biotechnology, Santa Cruz, CA, USA) were used at a concentration of 1:1000. The host of antibodies against TNFα and β-actin was mouse, while in the case of CB1, CB2 and Na^+^K^+^ATPase, the host was rabbit. Secondary antibodies labeled with alkaline phosphatase were used at a concentration of 1:1000 (Sigma-Aldrich, St. Louis, MO, USA). Protein bands visualization was made using the BCIP/NBT Liquid substrate system. The Versa Doc System and Quantity One software (Bio-Rad Laboratories Inc., Hercules, CA, USA) was applied for bands quantification. Level of each protein was expressed as the percentage of the protein intensity obtained in the control cells.

### 2.8. Examination of Zeta Potential

Zeta potential of the cell membrane of keratinocytes and fibroblasts was measured in live cells suspended in PBS and placed in a measuring vessel in a Zetasizer Nano ZS apparatus (Malvern Instruments, Malvern, UK). This apparatus uses a process called Dynamic Light Scattering (DLS) and Laser Doppler Velocimetry (LDV). The results were presented in the millivolts.

### 2.9. Statistical Analysis

The results presented in figures are expressed as the mean ± standard deviation (SD) for *n* = 3. The differences between means obtained for each experimental group were analyzed using multivariate analysis (one-way ANOVA), and *p*-values at least less than 0.05 were considered significant.

## 3. Results

### 3.1. Antioxidant Effect of Ascorbic Acid and Rutin

The obtained results indicated that rutin and ascorbic acid significantly prevented UVA and UVB-induced changes in the level of lipid mediators and endocannabinoid system functioning in skin fibroblasts and keratinocytes. This may be connected with changes in the antioxidant activity of cells caused by compounds with antioxidant properties. Despite the fact that rutin showed, in an experiment outside the biological system, two times weaker antioxidant properties than ascorbic acid (Figure 1A), both compounds significantly reduced UV-induced ROS generation in keratinocytes and fibroblasts (Figure 1B,C). A similar effect was also observed in the case of the simultaneous use of ascorbic acid and rutin; however, the stronger action of the mixture compared to the individual compounds was observed only when following UVA radiation.

### 3.2. Effect of Ascorbic Acid and Rutin on Lipid Metabolism

**Figure 2:**
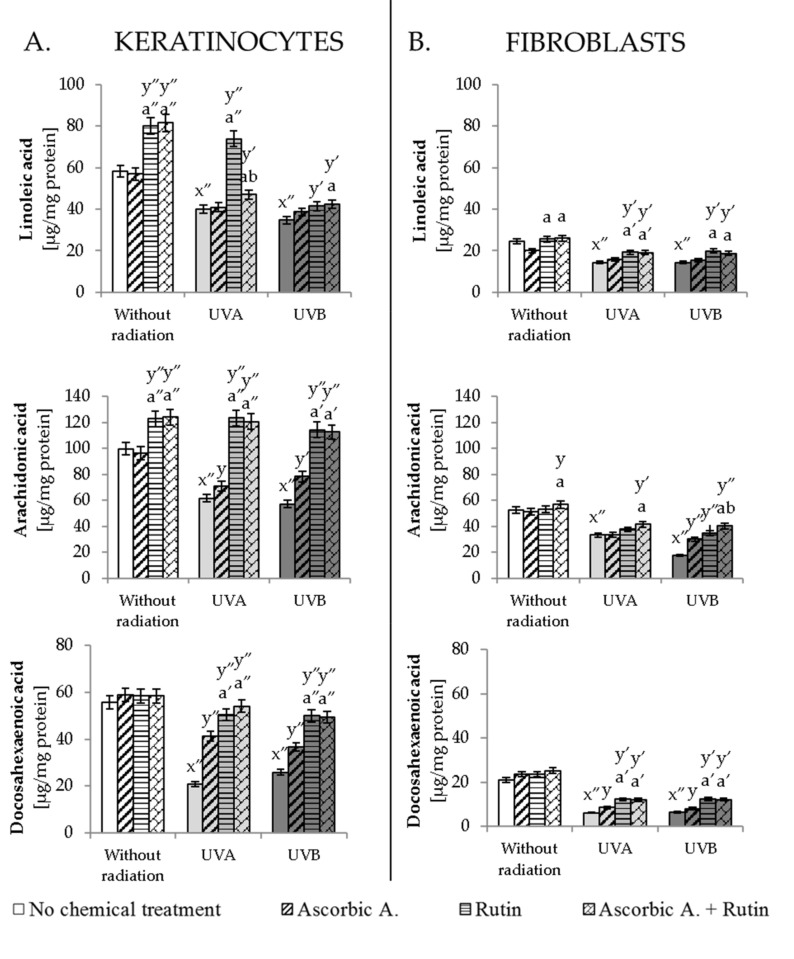
The level of selected phospholipid fatty acids in keratinocytes (**A**) and fibroblasts (**B**) exposed to UVA (30 J/cm^2^ and 20 J/cm^2^, respectively) and UVB irradiation (60 mJ/cm^2^ and 200 mJ/cm^2^, respectively) and treated with rutin (25 µM) and ascorbic acid (100 µM). Mean values ± SD of three independent experiments are presented. x significant differences vs. non-treated group, respectively (x″ for *p* < 0.0005); y significant differences vs. group without chemical treatment, respectively (y for *p* < 0.05; y′ for *p* < 0.005, y″ for *p* < 0.0005); a significant differences vs. ascorbic acid-treated group, respectively (a for *p* < 0.05; a′ for *p* < 0.005, a″ for *p* < 0.0005); b significant differences vs. rutin-treated group, respectively (b for *p* < 0.05).

UV-enhanced ROS generation conducive to the formation of oxidative stress directly influenced the composition of phospholipid fatty acids building cell membranes. UVA and UVB in keratinocytes and fibroblasts induced a statistically significant decrease in the level of linoleic acid (LA), arachidonic acid (AA) and docosahexaenoic acid (DHA), which was strongly prevented by the ascorbic acid and rutin used together (Figure 2). This action did not completely prevent the effects of UV radiation and did not restore phospholipid fatty acids levels close to control cells; however, ascorbic acid and rutin used together led to two times higher levels of AA and DHA compared to the UVA- or UVB-irradiated keratinocytes or fibroblasts. What deserves special attention is the fact that a similar protective effect was also observed for rutin but not for ascorbic acid-treated cells following UV irradiation.

**Figure 3:**
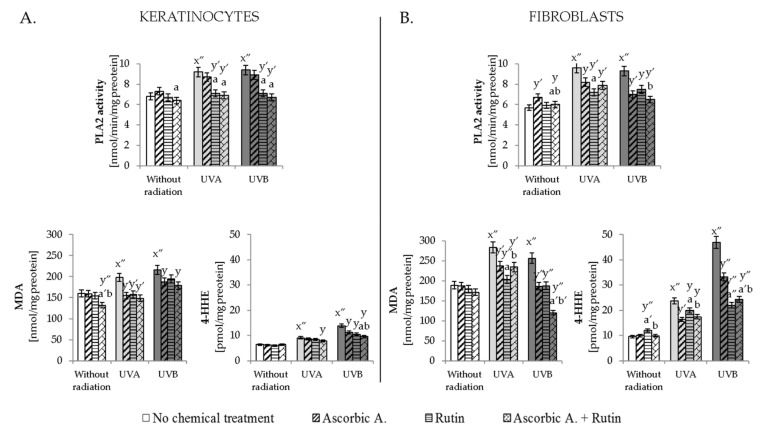
The activity of lipid metabolizing enzyme phospholipase A2 (PLA2) and levels of lipid peroxidation products (MDA and 4-HHE) in keratinocytes (**A**) and fibroblasts (**B**) exposed to UVA (30 J/cm^2^ and 20 J/cm^2^, respectively) and UVB irradiation (60 mJ/cm^2^ and 200 mJ/cm^2^, respectively) and treated with rutin (25 µM) and ascorbic acid (100 µM). Mean values ± SD of three independent experiments are presented. x significant differences vs. non-treated group, respectively (x″ for *p* < 0.0005); y significant differences vs. group without chemical treatment, respectively (y for *p* < 0.05; y′ for *p* < 0.005, y″ for *p* < 0.0005); a significant differences vs. ascorbic acid-treated group, respectively (a for *p* < 0.05; a′ for *p* < 0.005, a″ for *p* < 0.0005); b significant differences vs. rutin-treated group, respectively (b for *p* < 0.05; b′ for *p* < 0.005).

The level of phospholipid fatty acids is closely related to the activity of the enzyme involved in the lipid metabolism-PLA2. UV radiation increased the activity of this enzyme (Figure 3). Rutin significantly prevented this change, while ascorbic acid showed no action in relation to the activity of PLA2 in UV-irradiated keratinocytes. The combination of ascorbic acid and rutin in most cases led to the lower PLA2 activity than ascorbic acid used separately. However, this mixture induced a significantly stronger effect than rutin only in PLA2 activity in UVB-irradiated fibroblasts.

As a result of UV-induced ROS generation and enzymatic degradation of lipids in UVA- and UVB-irradiated skin cells, an enhanced level of lipid peroxidation products, including MDA and 4-HHE, was observed (Figure 3). In both experimental cell lines, the level of 4-HHE was more enhanced by UV radiation than in the case of UV-increased MDA levels. While MDA level was enhanced by 30–45% in keratinocytes and by 40–50% in fibroblasts, the 4-HHE level was increased by 30–90% in keratinocytes and 2–5 times in fibroblasts following UVA and UVB irradiation, respectively. Ascorbic acid, as well as rutin, to varying degrees, decreased the lipid peroxidation products following UV irradiation, except 4-HHE in UVA-irradiated keratinocytes. However, combined ascorbic acid and rutin treatment significantly protected lipids against peroxidation stronger than compounds used separately only in the case of 4-HHE in UVA/B-irradiated keratinocytes and MDA in UVB-irradiated fibroblasts.

### 3.3. Effect of Ascorbic Acid and Rutin on Endocannabinoid System

**Figure 4:**
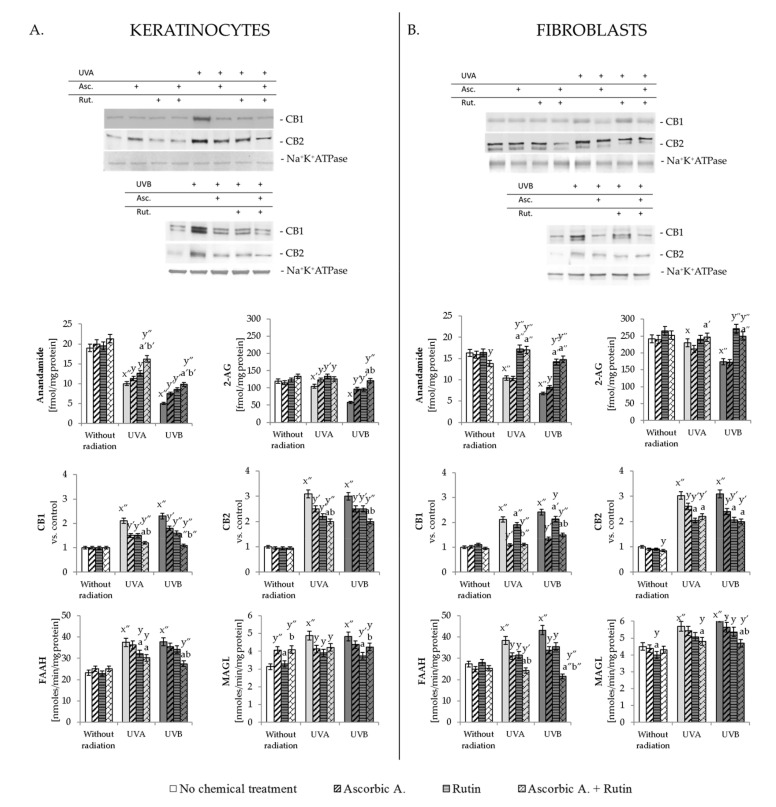
The changes in endocannabinoid system measured as the level of endocannabinoids (anandamide and 2-AG), expression of their receptors (CB1 and CB2) and activity of their degrading enzymes (FAAH and MAGL) in keratinocytes (**A**) and fibroblasts (**B**) exposed to UVA (30 J/cm^2^ and 20 J/cm^2^, respectively) and UVB irradiation (60 mJ/cm^2^ and 200 mJ/cm^2^, respectively) and treated with rutin (25 µM) and ascorbic acid (100 µM). Mean values ± SD of three independent experiments are presented. x significant differences vs. non-treated group, respectively (x for *p* < 0.05; x′ for *p* < 0.005, x″ for *p* < 0.0005); y significant differences vs. group without chemical treatment, respectively (y for *p* < 0.05; y′ for *p* < 0.005, y″ for *p* < 0.0005); a significant differences vs. ascorbic acid-treated group, respectively (a for *p* < 0.05; a′ for *p* < 0.005, a″ for *p* < 0.0005); b significant differences vs. rutin-treated group, respectively (b for *p* < 0.05; b′ for *p* < 0.005, b″ for *p* < 0.0005); + indicates the cells treatment in each case.

Presented in Figure 4, results clearly show that UVA and UVB radiation influenced the action of the endocannabinoid system in skin fibroblasts and keratinocytes. It is demonstrated by decreased levels of endocannabinoids AEA and 2-AG that both cell lines were decreased by UVB radiation stronger than by UVA. In the case of keratinocytes, ascorbic acid, as well as rutin, prevented UV-induced changes; however, the strongest protective effect was observed when both compounds were used together. Different situations took place for UV-irradiated fibroblasts, where cells treatment with ascorbic acid did not cause changes in the AEA and 2-AG levels, while rutin or ascorbic acid with rutin treatment significantly prevented UV-induced lowering their levels. At the same time, UV radiation increases CB1/2 expression in skin cells membrane, which was also in various degrees prevented by the ascorbic acid or/and rutin. Again for UV-irradiated keratinocytes, combined ascorbic acid and rutin treatment showed the strongest protective effect for both types of receptors, while UV-irradiated fibroblasts reacted in more varied ways. It was observed that in UVA or UVB-irradiated fibroblasts, ascorbic acid (and ascorbic acid + rutin) most strongly decreased the level of the CB1 receptor, while in the case of the CB2 receptor, rutin (and ascorbic acid + rutin)-treated fibroblasts caused the strongest decrease in the expression of this protein following cells irradiation with UVA as well as UVB.

On the other hand, the decreased level of endocannabinoids also depends on the activity of enzymes that degrade them and, as shown in the obtained results, UV radiation was able to significantly enhance FAAH and MAGL activity (around 60% compared to the control cells). Rutin and ascorbic acid, used separately or together slightly but with statistical significance, reduced the FAAH and MAGL activity in the case of UV-irradiated keratinocytes. However, the protective effect of these compounds was much more visible in the case of FAAH activity in UV-irradiated fibroblasts, where used separately decreased FAAH activity by about 20%, but used together led to a reduction of up to 55% (Figure 4).

### 3.4. Ascorbic Acid and Rutin Protection against UV-Induced Inflammation and Cell Death

**Figure 5:**
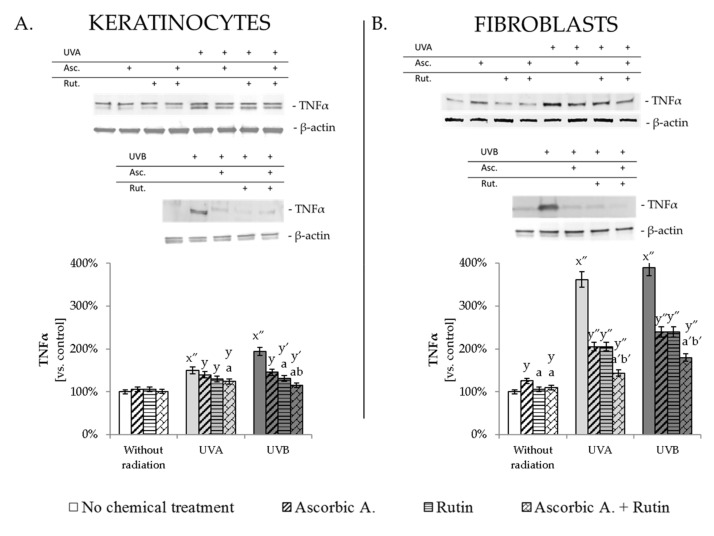
The changes in pro-inflammatory factor TNFα in keratinocytes (**A**) and fibroblasts (**B**) exposed to UVA (30 J/cm^2^ and 20 J/cm^2^, respectively) and UVB irradiation (60 mJ/cm^2^ and 200 mJ/cm^2^, respectively) and treated with ascorbic acid (Asc., 100 µM) and rutin (Rut., 25 µM). Mean values ± SD of three independent experiments are presented. x significant differences vs. non-treated group, respectively (0.005, x″ for *p* < 0.0005); y significant differences vs. group without chemical treatment, respectively (y for *p* < 0.05; y′ for *p* < 0.005, y″ for *p* < 0.0005); a significant differences vs. ascorbic acid-treated group, respectively (a for *p* < 0.05; a′ for *p* < 0.005); b significant differences vs. rutin-treated group, respectively (b for *p* < 0.05; b′ for *p* < 0.005); + indicates the cells treatment in each case.

**Figure 6:**
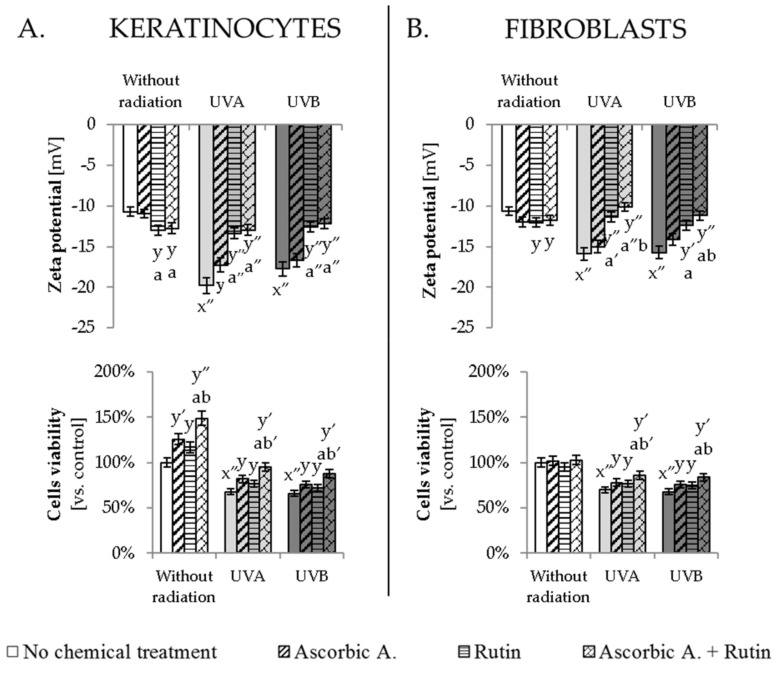
The changes in the Zeta potential and cells viability of keratinocytes (**A**) and fibroblasts (**B**) exposed to UVA (30 J/cm^2^ and 20 J/cm^2^, respectively) and UVB irradiation (60 mJ/cm^2^ and 200 mJ/cm^2^, respectively) and treated with ascorbic acid (Asc., 100 µM) and rutin (Rut., 25 µM). Mean values ± SD of three independent experiments are presented. x significant differences vs. non-treated group, respectively (x″ for *p* < 0.0005); y significant differences vs. group without chemical treatment, respectively (y for *p* < 0.05; y′ for *p* < 0.005, y″ for *p* < 0.0005); a significant differences vs. ascorbic acid-treated group, respectively (a for *p* < 0.05; a′ for *p* < 0.005, a″ for *p* < 0.0005); b significant differences vs. rutin-treated group, respectively (b for *p* < 0.05; b′ for *p* < 0.005).

Described changes induced by UV radiation in the endocannabinoid system resulted in increased pro-inflammatory factors and enhanced cells viability. UVA and UVB radiation led to enhanced pro-inflammatory factor TNFα in keratinocytes by about 50% and 90%, respectively, while in fibroblasts, this increase was 3.5–4 times following UVA and UVB. Ascorbic acid and rutin used separately or together decreased UV-induced TNFα expression in both tested cell lines; however, the effects of these protective compounds were more visible for fibroblasts, where ascorbic acid, as well as rutin, used separately, decreased TNFα level by 40% and by 55% when cells were treated with both compounds together (Figure 5, Supplementary Figure S1). Moreover, UV-induced changes in phospholipid composition influenced the Zeta potential of the cellular membrane, which was observed even as two times stronger membrane potential in UV-irradiated keratinocytes, and around 50% stronger membrane potential in UV-irradiated fibroblasts (Figure 6). The cells treated with rutin or ascorbic acid + rutin following UVA/B irradiation significantly prevented these changes and restored Zeta potential levels even close to those of control cells. As a result, all these described changes contributed to UV-induced reduction in cells viability, estimated at 70% compared to the non-treated keratinocytes and fibroblasts (Figure 6). Ascorbic acid and rutin increased the viability of non-irradiated keratinocytes by 20% when used separately and by 50% in the case of combined treatment. Tested compounds also enhanced both cell lines viability following UV irradiation, which was observed at the level of around 10% increase in viability when compounds were used separately, and 15% for ascorbic acid with rutin treatment.

## 4. Discussion

### 4.1. Rutin and Ascorbic Acid Effect on UV-Induced Changes in Phospholipid Composition

The proper functioning of the skin is inextricably linked with the proper functioning of all its compartments, which is conditioned by the physiological structure and function of its essential compounds, including proteins, lipids, carbohydrates and nucleic acids. Despite the variety of fulfilled by these molecules functions, such as enzymatic activity, signal transduction, genetic information management or structural functions, lipids require the greatest attention in the case of skin cells. Lipids not only build cell membranes but their derivatives are involved in various cellular processes in the cytoplasm, including signal transduction. Disturbances in membrane phospholipid composition and structure are associated with the failure of the natural action of many signaling pathways and physiologic processes, resulting in metabolic disorders and even individual cell death. It can be visualized as a significant reduction in the condition of the skin, but simultaneously, it can also be associated with dangerous effects for the entire body [2]. Additionally, being a part of the intercellular matrix in the epidermis, lipid derivatives maintain skin consistency and prevent water loss from the body [33]. Therefore, in this study, the changes in lipid metabolism and the structural and metabolic consequences of these changes induced by UV radiation, as well as the natural way of preventing their formation, were assessed.

UVA/UVB radiation that penetrates epidermis and dermis is known as a factor that, through photosensitizers activation, as well as by direct action on cellular metabolism, induces free radicals generation in skin cells [3,34]. The accompanying failure of the antioxidant system leads to oxidative stress, which is the direct reason for disturbances in phospholipid metabolism, including their enzymatic- as well as ROS-dependent oxidation. As a result, the decrease in the phospholipid fatty acids, especially polyunsaturated (PUFAs), including LA, AA and DHA, is observed, which suggests their release from the membrane in a free form. Free PUFAs in human skin may arbitrate the UV effect, especially in terms of pro-inflammatory reactions and sunburn [35]; therefore, in UV-irradiated cells, the enzymes responsible for the metabolism of phospholipids, including PLA2, are also upregulated. Under oxidative stress conditions and consequently after PLA2 activation, the level of phospholipid fatty acids is reduced, which is accompanied by the release of oxidative cyclization products, such as isoprostanes as well as free fatty acids, which undergo peroxidation to low molecular weight reactive aldehydes such as MDA and 4-HHE [36]. As a result, there are modifications to the structure and functions of cell membranes, including disturbances in membrane permeability. However, the aforementioned aldehydes, by binding to proteins, may also affect intracellular signaling as well as the antioxidant capacity of cells, and through interaction with DNA, they may even lead to neoplastic transformation [5,37].

Therefore, naturally occurring antioxidants such as ascorbic acid and rutin have been proposed in the search for protecting skin cells against the oxidative effects of UV radiation. Their antioxidant action as effective free radical scavengers has been described previously [15] and is also shown in this study. In all the above cases, ascorbic acid shows a stronger antioxidant effect than rutin; however, the combination of these components always leads to enhanced mutual antioxidant properties. What deserves careful attention is the fact that examined skin cell types under conditions without exposure to UV radiation react differently to ascorbic acid and rutin used separately or together, which is most evident in the viability of these cells. The hypothesis explaining this observation is based on the evolutionary adaptation of keratinocytes. Proliferation, growth and differentiation leading to keratinization and death of keratinocytes under physiological conditions are processes that occur many times faster than in the case of skin fibroblasts [38]. Therefore, as well as referring to the external location of these cells, their higher viability may be the result of the supplementation with cytoprotective compounds, ensuring the protection of their genetic material transferred to daughter cells [39].

Additionally, in this study, a protective potential of ascorbic acid and rutin is shown in relation to phospholipid PUFAs in UV-irradiated keratinocytes and fibroblasts. Previous data indicate that in the case of rutin, this protective action is time-dependent and starts immediately after cell supplementation with rutin [40]. This is also connected with the activity of enzymes degrading phospholipids, such as PLA2, as well metabolizing fatty acids, such as cyclooxygenases (COXs), lipoxygenases (LOXs) or diacylglycerol lipase (DAGL), which lead to the formation of lipid mediators, including reactive aldehydes, eicosanoids and endocannabinoids [41,42]. All of these enzyme activities are enhanced by ROS and strongly inhibited by antioxidants action [42,43]. Rutin has also been known for many years as a powerful inhibitor of PLA2, which results in phospholipid polyunsaturated fatty acids protection and prevention against inflammatory reactions and apoptosis [40,44,45]. This response is also observed for UV-irradiated keratinocytes and fibroblasts in this study, whether the cells were treated only with rutin or with ascorbic acid and rutin. However, ascorbic acid is not able to reduce the PLA2 activity in UV-irradiated keratinocytes, which is connected with the role of ascorbic acid in proper keratinocytes differentiation, aging and keratinization [46].

Moreover, as a result of the antioxidant properties of ascorbic acid and rutin, the decrease in ROS generation and lipid peroxidation products (MDA and 4-HHE) levels are observed. However, the combined action of ascorbic acid and rutin is more effective in the case of cells irradiated with UVA than those exposed to UVB, which confirms the stronger pro-oxidative effect of this radiation as well as its direct influence on, among others, the degradation of rutin [47]. On the other hand, the comparison of the protective effect of ascorbic acid and rutin on lipid peroxidation processes in various types of cells indicates that the compounds used are more effective for UV-irradiated fibroblasts than for keratinocytes. This may be partly related to the greater natural resistance of keratinocytes to environmental factors [48], which in the case of UV radiation is manifested, among others, by lower ROS generation and reduced activity of phospholipid-degrading enzymes, which results in smaller levels of lipid peroxidation products than in fibroblasts, which require additional external protection.

The comparison of the described effects of ascorbic acid and rutin shows the advantages of the action of these compounds not only as independent antioxidants but also indicates different ranges of their activity, which gives positive results in the case of UV-irradiated skin cells and mixed treatment.

### 4.2. Effect of Rutin and Ascorbic Acid on the UV-Modified Endocannabinoid System Activity

One of the groups of products formed in the conditions of physiological enzymatic metabolism of membrane phospholipids is endocannabinoids. The main known endocannabinoids are AEA and 2-AG, which act as agonists of G-protein-coupled receptors, including cannabinoid receptors (CB1/2). As a result, many pathways important for proper cells functioning can be activated, including activation of focal adhesion kinase (FAK), mitogen-activated protein kinase (MAPK), stimulation of nitric oxide synthase (NOS), regulation of ion currents and induction of immediate early genes [49]. Therefore, the significant decrease in the level of AEA and 2-AG of skin cells after UV irradiation, observed in this study, as well as in a previous report [3], indicates a strong disturbance in the entire endocannabinoid system, resulting in a disturbance of indicated pathways. The UV-induced decrease in AEA and 2-AG levels is due to the enhanced activity of endocannabinoids degrading enzymes FAAH and MAGL [50,51]. FAAH is the major enzyme that degrades AEA, while MAGL hydrolyzes 2-AG. The increased activity of these enzymes is characteristic for various pathological conditions, including exposure to harmful environmental factors (UV radiation) [3], but also disorders of the nervous system (pain, inflammation, anxiety, depression), neuroinflammatory and neoplastic diseases [52,53,54]. Therefore, the inhibition of the activity of these enzymes by ascorbic acid and rutin may become the subject of research not only in terms of the protection of skin cells but also in the assessment of therapeutic factors in relation to developing diseases of this organ. So far, plant extracts containing polyphenols, including rutin, have been described as preparations that strongly reduce the activity of FAAH and MAGL [55,56], which has not been previously shown for ascorbic acid.

The decreased level of AEA and 2-AG may be due to their binding to CB1/2 receptors, the expression of which is enhanced by UV radiation. As mentioned earlier, the specific activity of CB1 is related to the generation of ROS and TNFα, which may result in an antioxidant and anti-inflammatory response of cells, while CB2 activation leads to a reduction of both ROS and TNFα [10]. Therefore, the activation of both cannabinoid receptors observed in this study may be directly related to their participation in the modification of cellular redox balance and inflammation under the influence of UV radiation, as well as the simultaneous reaction of skin cells to this pathology. Particularly noteworthy is the fact that cells, in response to UV radiation, activate extremely strong CB2 receptors responsible for restoring physiological conditions. Regardless of the skin cell type or the UV wavelength used, ascorbic acid and rutin partially or completely prevent UV-induced changes in endocannabinoid levels or receptor expression. Similar results were previously observed for these compounds when used separately [16,17]. Rutin has also been shown to be a molecule that can so efficiently modulate the expression of CB1 in various stress model systems that the physiological levels of this receptor are restored regardless of the conditions [57].

The results presented in this study indicate that the combined use of ascorbic acid and rutin tends to restore many parameters to values comparable to those of the control cells. Therefore, the cooperation in the action of ascorbic acid and rutin favors the elimination of the harmful environmental factor effects, which was also previously observed for the combined use of ascorbic acid and rutin for UV-irradiated skin cells in the case of comprehensive proteomic analyses [19,20,21]. Both earlier and current studies allow for the hypothesis that the increased activity of both compounds is the result of the support by ascorbic acid in rutin membrane penetration, as well as mutual protection of rutin and ascorbic acid antioxidants against oxidation and degradation [15,58,59]. This allows for the maintenance of the pool of reduced antioxidants both in the cytoplasm and in the lipid fraction of cells, which indicates the justified use of rutin and ascorbic acid in a duet.

### 4.3. Protective Effect of Rutin and Ascorbic Acid against the UV-Induced Inflammation

As described above, UV-induced changes in lipid metabolism, leading to an increased level of lipid peroxidation products and endocannabinoid system activation, resulting in an increased inflammatory process. It is manifested by increased TNFα level, which is the basic pro-inflammatory factor expression under the skin cell response to UV radiation [60]. The action of TNFα is strictly connected with the NFκB factor that, following TNFα receptors activation, is released from its inhibitory complex with IκB, and in turn induces the expression of TNFα, as well as other pro-inflammatory cytokines [61]. Lipid peroxidation products upregulated by UV radiation have been reported to interact with NFκB/IκB in the induction of TNFα [5]. Additionally, the increased level of CB1 has been connected with the stimulation of TNFα expression [62]. Therefore, the protective effects of rutin and ascorbic acid used separately, as well as their mutually enhanced effect, are also reflected in lowering the level of TNFα in UV-irradiated keratinocytes and fibroblasts. Moreover, UV-induced disturbances in the lipid metabolism, confirmed by changes in the composition of phospholipid fatty acids, significantly change the Zeta potential of cells membrane, which has an impact on the transporters activity and membrane permeability, decreasing cells viability [40]. Additionally, in this case, ascorbic acid and rutin, by influencing membrane phospholipids metabolism, effectively protect skin cells against metabolism disruption and decreased viability following UV irradiation. This observation in the case of Zeta potential is based mainly on the rutin properties, also showed before [40], which accumulate mainly in membranes; however, its protective and antioxidant action is strongly supported by ascorbic acid, thus explaining the need and advantages of using these compounds together.

## 5. Conclusions

In conclusion, it can be suggested that ascorbic acid and rutin mutually support their protective effect in relation to the various types of skin cells regardless of the wavelength of UV radiation to which they have been exposed. This action is based not only on their combined antioxidant capacities that protect against lipid peroxidation but also decrease the UV-induced inflammation by direct interaction with the endocannabinoid system. Moreover, the comparison of the described effects of ascorbic acid and rutin indicates different ranges of their activity, the mutual support of which could give positive results in the case of UV-irradiated skin cells treated with a mixture of both compounds.

## Data Availability

Data are contained within the article and supplementary material.

## Supplementary Materials

The following are available online at https://www.mdpi.com/article/10.3390/antiox10081260/s1, Figure S1: Full pictures of Western blot analysis of endocannabinoids receptors CB1/CB2 and pro-inflammatory factor TNFα expression in keratinocytes and fibroblasts exposed to UVA (30 J/cm^2^ and 20 J/cm^2^, respectively) and UVB irradiation (60 mJ/cm^2^ and 200 mJ/cm^2^, respectively) and treated with ascorbic acid (Asc., 100 µM) and rutin (Rut., 25 µM).
