# Supplementary material for: Exogenous Antioxidants Impact on UV-Induced Changes in Membrane Phospholipids and the Effectiveness of the Endocannabinoid System in Human Skin Cells
Source: Antioxidants (Basel). 2021 Aug 6;10(8):1260. doi: 10.3390/antiox10081260 (PMC8389309; doi:10.3390/antiox10081260)
Supplement: Supplementary file 1 [file antioxidants-10-01260-s001.zip › antioxidants-1317997-supplementary.pdf]

Supplementary Figure S1

Full pictures of Western blot analysis of endocannabinoids receptors CB1/CB2 and pro-inflammatory factor TNFα expression in keratinocytes and fibroblasts exposed to UVA (30 J/cm^2 and 20 J/cm^2, respectively) and UVB irradiation (60 mJ/cm^2 and 200 mJ/cm^2, respectively) and treated with ascorbic acid (Asc., 100 μM) and rutin (Rut., 25 μM).

<u>KERATINOCYTES</u>

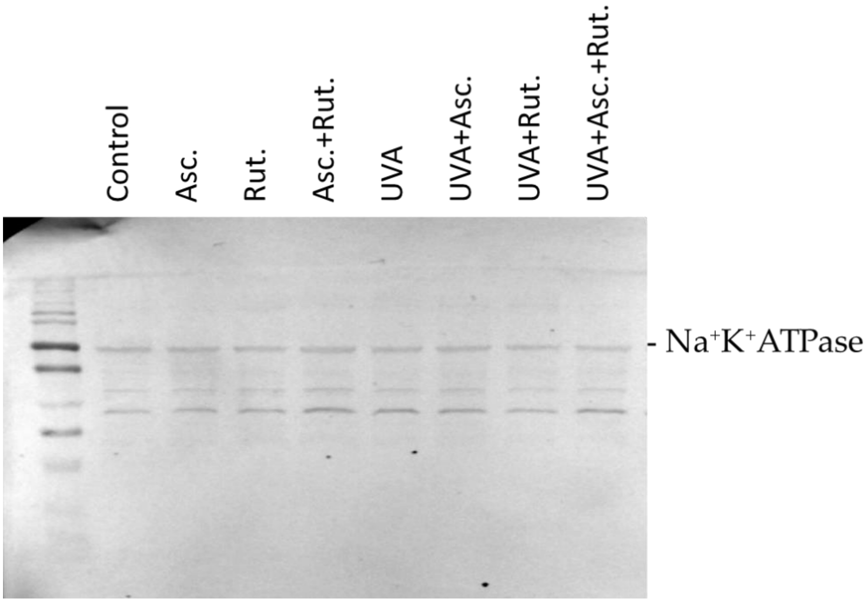

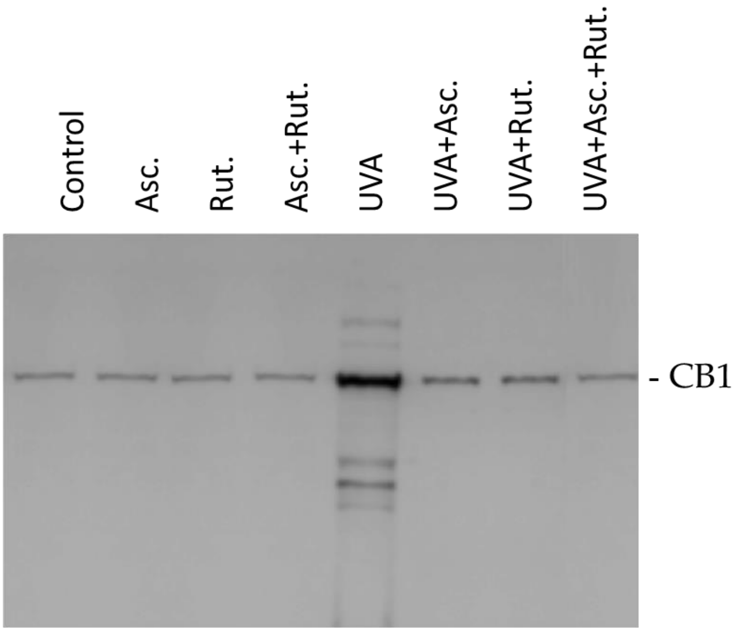

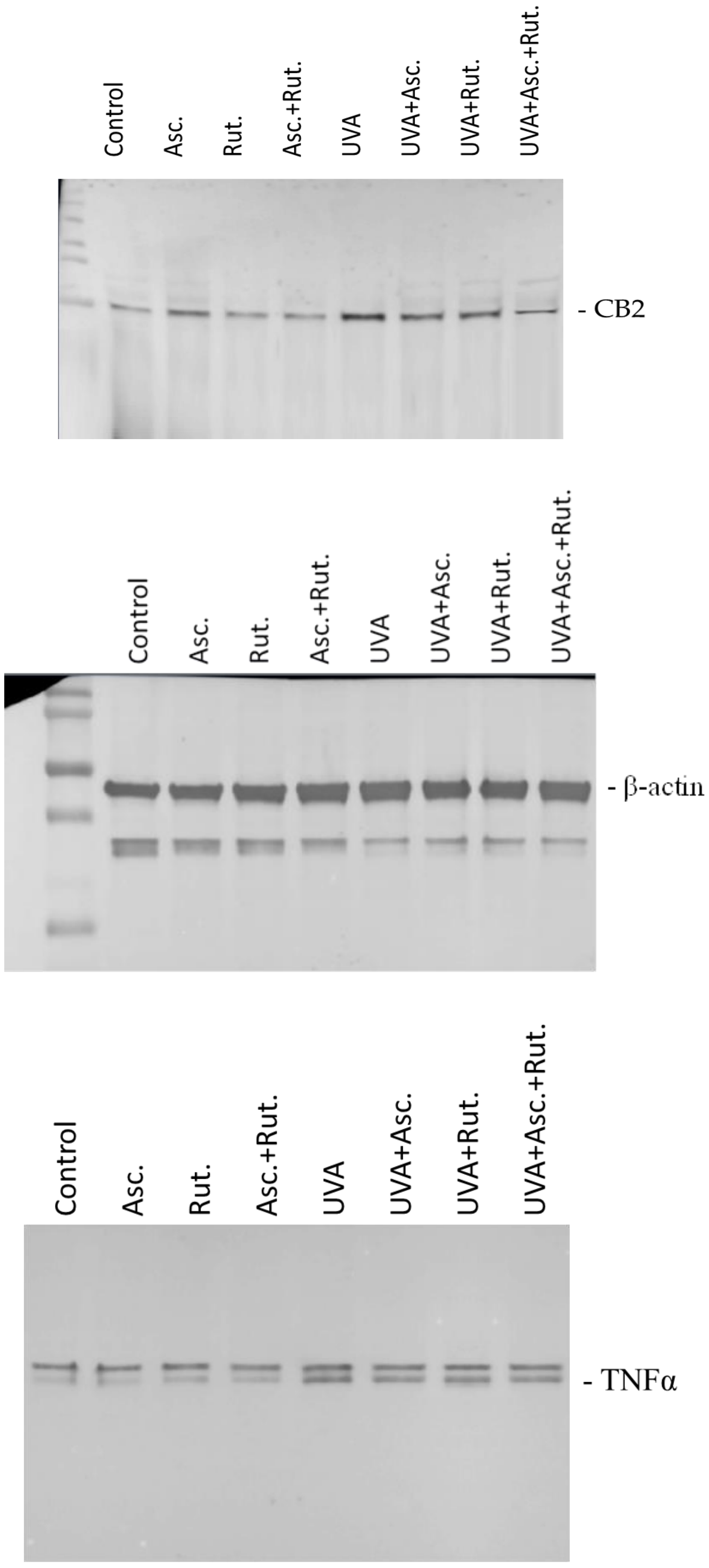
Control
Asc.
Rut.
Asc.+Rut.
UVA
UVA+Asc.
UVA+Rut.
UVA+Asc.+Rut.
- CB2
Control
Asc.
Rut.
Asc.+Rut.
UVA
UVA+Asc.
UVA+Rut.
UVA+Asc.+Rut.
- β-actin
Control
Asc.
Rut.
Asc.+Rut.
UVA
UVA+Asc.
UVA+Rut.
UVA+Asc.+Rut.
- TNFα

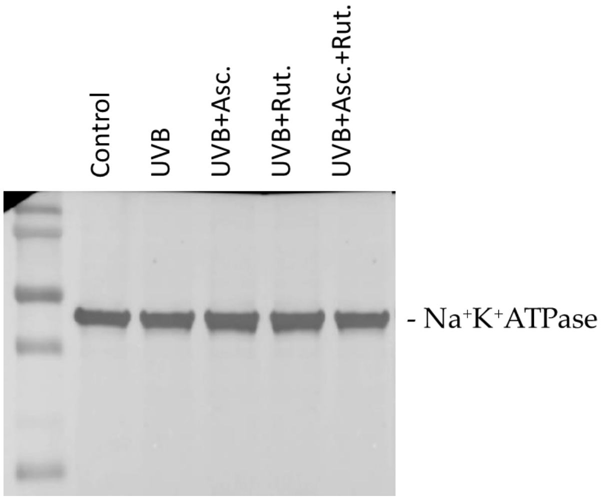
Control
UVB
UVB+Asc.
UVB+Rut.
UVB+Asc.+Rut.
- Na+K+ATPase

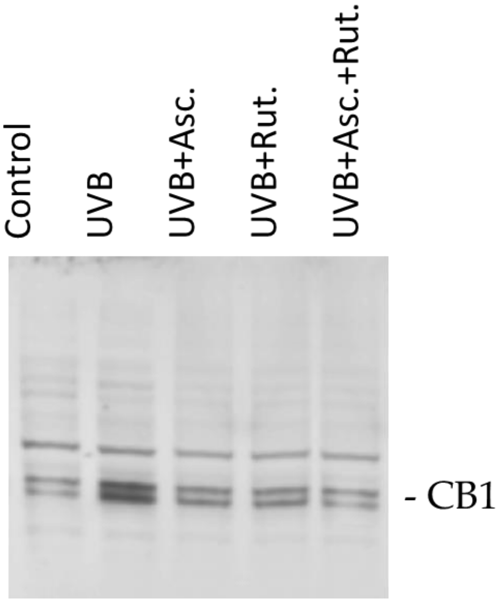
Control
UVB
UVB+Asc.
UVB+Rut.
UVB+Asc.+Rut.
- CB1

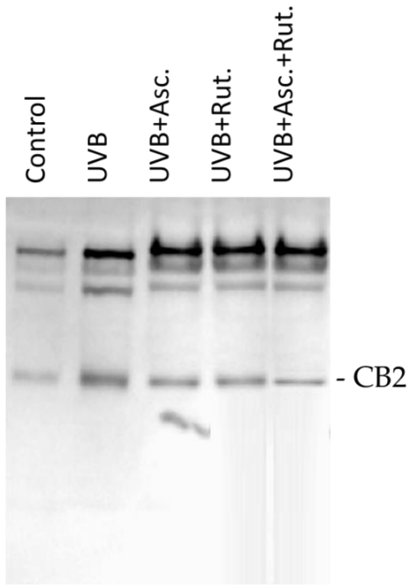
Control
UVB
UVB+Asc.
UVB+Rut.
UVB+Asc.+Rut.
- CB2

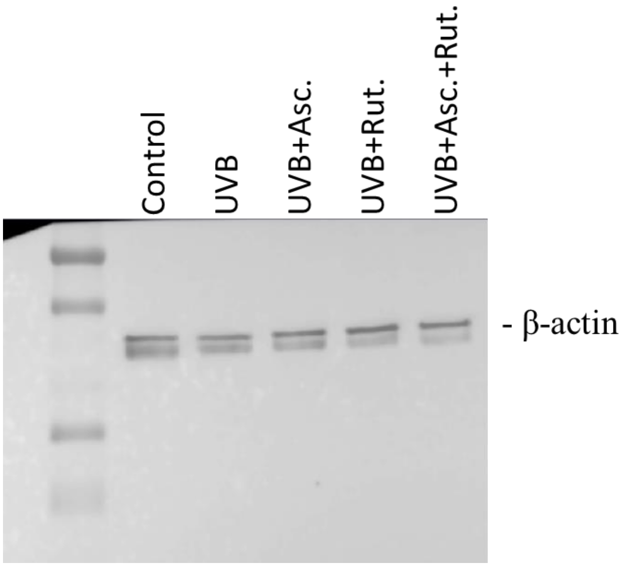
Control
UVB
UVB+Asc.
UVB+Rut.
UVB+Asc.+Rut.
- β-actin

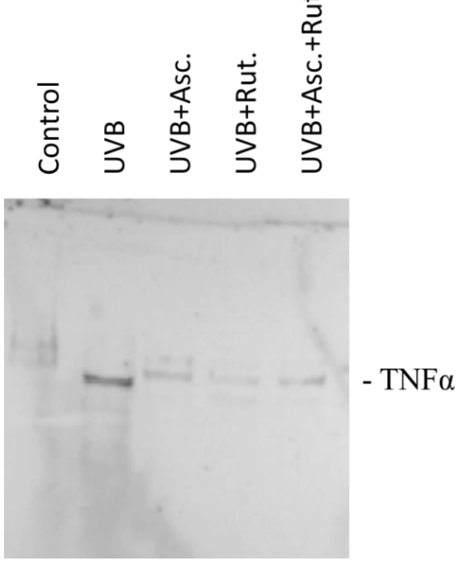
Control
UVB
UVB+Asc.
UVB+Rut.
UVB+Asc.+Rut.
- TNFα

FIBROBLASTS

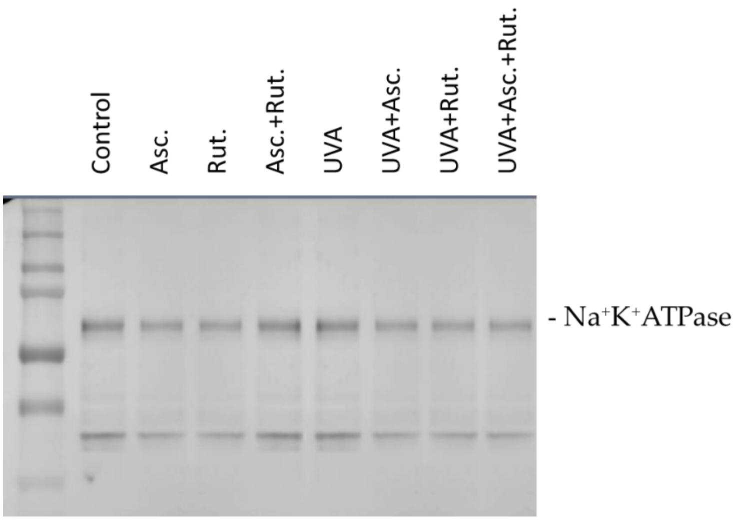

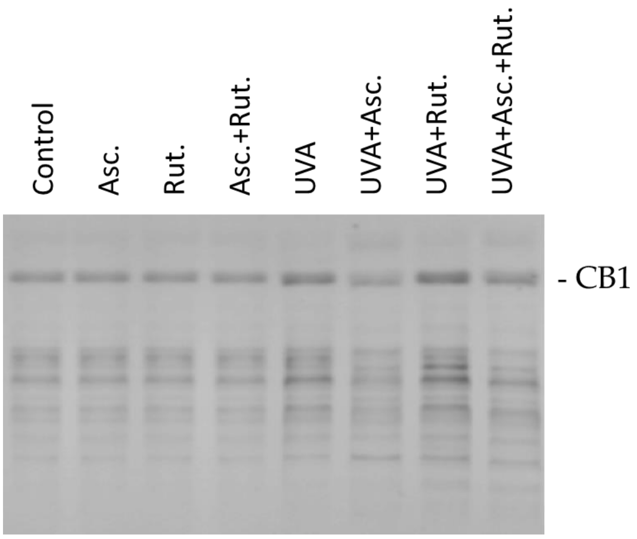

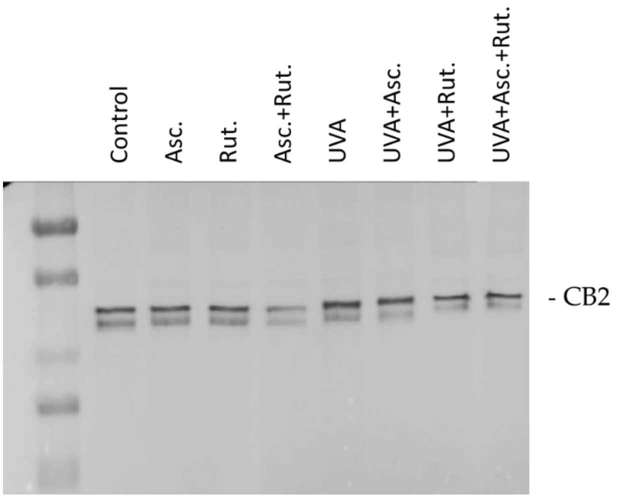

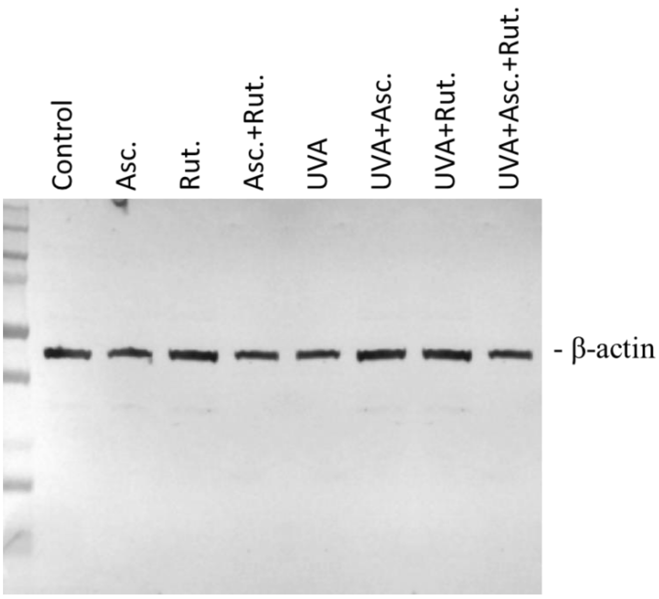
Control
Asc.
Rut.
Asc.+Rut.
UVA
UVA+Asc.
UVA+Rut.
UVA+Asc.+Rut.
- β-actin

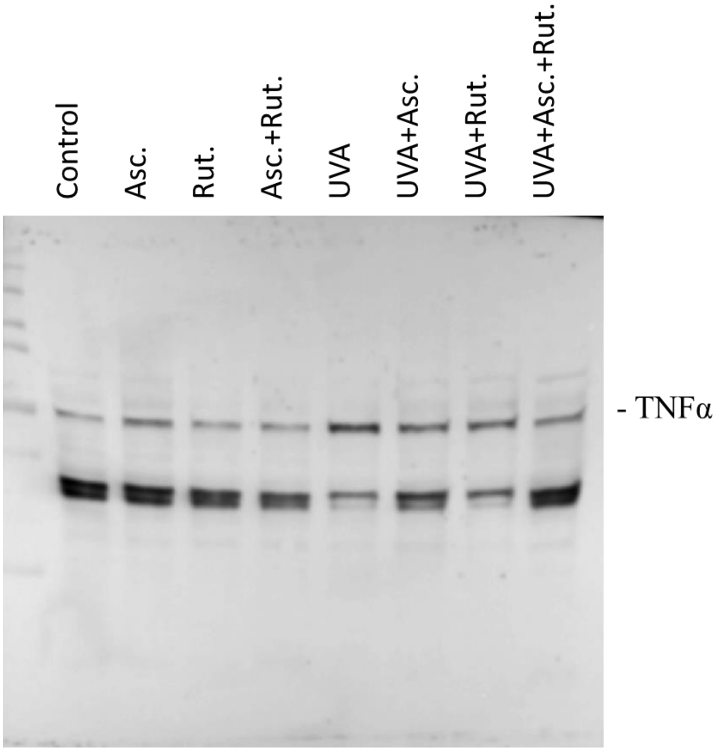
Control
Asc.
Rut.
Asc.+Rut.
UVA
UVA+Asc.
UVA+Rut.
UVA+Asc.+Rut.
- TNFα

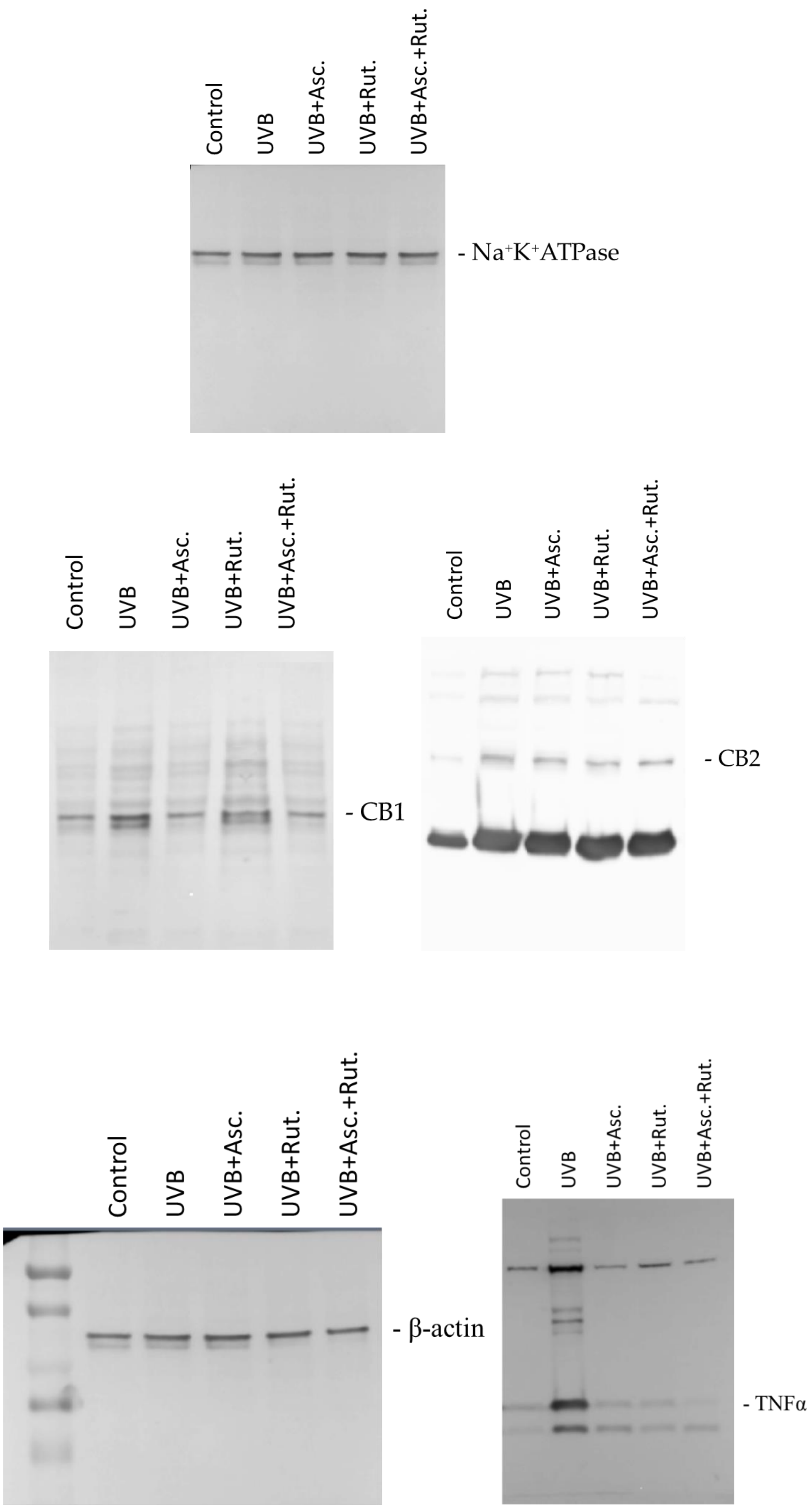
Control
UVB
UVB+Asc.
UVB+Rut.
UVB+Asc.+Rut.
- Na+K+ATPase
Control
UVB
UVB+Asc.
UVB+Rut.
UVB+Asc.+Rut.
- CB1
Control
UVB
UVB+Asc.
UVB+Rut.
UVB+Asc.+Rut.
- CB2
Control
UVB
UVB+Asc.
UVB+Rut.
UVB+Asc.+Rut.
- β-actin
Control
UVB
UVB+Asc.
UVB+Rut.
UVB+Asc.+Rut.
- TNFα
